# Matrix metalloproteinase 12 modulates high-fat-diet induced glomerular fibrogenesis and inflammation in a mouse model of obesity

**DOI:** 10.1038/srep20171

**Published:** 2016-01-29

**Authors:** Honglin Niu, Ying Li, Haibin Li, Yanqing Chi, Minghui Zhuang, Tao Zhang, Maodong Liu, Lei Nie

**Affiliations:** 1 Department of Nephrology, Third Hospital of Hebei Medical University, Shijiazhuang, 050051, China; 2 Key Laboratory of Kidney Diseases of Hebei Province, Shijiazhuang, 050071, China; 3 Department of Cardiology, Third Hospital of Hebei Medical University, Shijiazhuang, 050051, China; 4 Department of Nephrology, First Central Hospital of Baoding, Baoding, 071000, China; 5 Key Laboratory of Medical Biotechnology of Hebei Province and Key Laboratory of Neural and Vascular Biology of Ministry of Education, Hebei Medical University, Shijiazhuang, 050017, China; 6 Department of Biochemistry and Molecular Biology, College of Basic Medicine, Hebei Medical University, Shijiazhuang, 050017, China

## Abstract

Obesity-induced kidney injury contributes to albuminuria, which is characterized by a progressive decline in renal function leading to glomerulosclerosis and renal fibrosis. Matrix metalloproteinases (MMPs) modulate inflammation and fibrosis by degrading a variety of extracellular matrix and regulating the activities of effector proteins. Abnormal regulation of MMP-12 expression has been implicated in abdominal aortic aneurysm, atherosclerosis, and emphysema, but the underlying mechanisms remain unclear. The present study examined the function of MMP-12 in glomerular fibrogenesis and inflammation using *apo E*^−/−^ or *apo E*^−/−^*MMP-12*^−/−^ mice and maintained on a high-fat-diet (HFD) for 3, 6, or 9 months. *MMP-12* deletion reduced glomerular matrix accumulation, and downregulated the expression of NADPH oxidase 4 and the subunit-p67^phox^, indicating the inhibition of renal oxidative stress. In addition, the expression of the inflammation-associated molecule MCP-1 and macrophage marker-CD11b was decreased in glomeruli of *apo E*^−/−^*MMP-12*^−/−^ mice fed HFD. MMP-12 produced by macrophages infiltrating into glomeruli contributed to the degradation of collagen type IV and fibronectin. Crescent formation due to renal oxidative stress in Bowman’s space was a major factor in the development of fibrogenesis and inflammation. These results suggest that regulating MMP-12 activity could be a therapeutic strategy for the treatment of crescentic glomerulonephritis and fibrogenesis.

The global incidence of obesity is increasing; currently, there are >1 billion adults who are overweight, of which at least 300 million are clinically obese (i.e., body mass index >30 kg/m^2^). As an inflammatory disease, obesity is a major contributing factor in metabolic syndromes, cardiovascular disease, and diabetes^1^, and clinical and experimental studies have shown that it is an independent risk factor for chronic kidney disease (CKD) and end-stage renal disease^2^,^3^. Hyperglycemia, dyslipidemia, and oxidative stress-all pathological processes in obesity-induced kidney disease—contribute to albuminuria and progressive decline in renal function that ultimately lead to glomerulosclerosis and renal fibrosis^4^,^5^,^6^. The latter is characterized by excessive extracellular matrix (ECM) accumulation and occurs in virtually every type of CKD^7^. The current model of renal fibrogenesis describes a process analogous to the wound-healing response to injury. In response to renal injury and inflammation, resident kidney cells and infiltrating inflammatory monocytes/macrophages and T cells activate and produce toxic molecules such as reactive oxygen species (ROS) in addition to fibrogenic and inflammatory cytokines. These in turn activate mesangial cells, fibroblasts, and tubular epithelial cells that secrete ECM components^8^,^9^,^10^. Excessive fibrosis results in the permanent loss of normal kidney function^4^,^5^.

Matrix metalloproteinases (MMPs) comprise a family of more than 20 distinct zinc endopeptidases that degrade and remodel ECM components including collagens, proteoglycans, fibronectin (FN), and laminin (LN). MMPs also regulate the activities of effector proteins involved in inflammation and fibrosis^7^,^9^. Macrophage metalloelastase (MMP-12) was first identified as an elastolytic metalloproteinase secreted by inflammatory macrophages; it is now known to be expressed in hypertrophic osteoclasts, vascular smooth muscle cells, and some cancer cell types^11^,^12^,^13^,^14^. MMP-12 can degrade not only its major substrate elastin, but also targets other ECM components such as collagen IV, FN, LN, vitronectin, proteoglycan, chondroitin sulfate, and myelin basic protein^4^,^7^,^15^. Abnormal regulation of MMP-12 expression has been implicated in abdominal aortic aneurysm, atherosclerosis, and emphysema^16^,^17^.

Macrophages catalyze and generate superoxides and other ROS via membrane-associated NADPH oxidase (Nox)^18^,^19^. The Nox family has seven members (Nox1–5, Duox-1 and -2). Nox1, Nox2, and Nox4 are expressed in both mouse and human kidney; Nox3 is expressed in the fetal kidney of human and mouse and Nox5 is exclusively expressed in the human kidney but not in rodents. The Nox subunits p47^phox^, p67^phox^, and p22^phox^ are differentially expressed in the various renal cell subtypes. ROS generated by Nox proteins participate in signal transduction and cell cycling, but their overproduction can lead to oxidative stress, which is considered a major cause of renal injury and inflammation in diverse pathologies^18^,^20^,^21^. High-fat-diet (HFD) can exacerbate oxidative stress and inflammation and contribute to metabolic syndromes and renal lipid accumulation and injury^3^,^22^.

While human macrophages express a variety of MMPs, mouse macrophages predominantly produce MMP-12, along with relatively low levels of MMP-9. The role of MMPs of ECM degradation by MMPs in renal fibrosis-associated ECM degradation and inflammation is complex^7^,^23^, comprising both positive and negative effects on kidney disease progression^7^,^11^. The present study investigated the MMP-12 function in obesity-induced glomerular fibrogenesis and inflammation in mice lacking *apolipoprotein E* alone (*apo E*^−/−^)—which are predisposed to obesity—or both *apo E* and *MMP-12* (*apo E*^−/−^*MMP-12*^−/−^) that were maintained on HFD for 3, 6, or 9 months. MMP-12 released by macrophages infiltrating into glomeruli induced fibrogenesis and inflammation, while *MMP-12* deficiency reduced glomerular matrix accumulation and suppressed renal oxidative stress. In addition, expression of the inflammation-associated molecule monocyte chemoattractant protein-1 (MCP-1) and macrophage marker-CD11b was decreased in glomeruli of *apo E*^−/−^*MMP-12*^−/−^ mice. The results indicate that MMP-12 modulates glomerular fibrogenesis and inflammation in renal disease induced by HFD, and suggest that drugs that target MMP-12 can be effective in preventing CKD.

## Results

### MMP-12 deletion attenuates HFD-induced renal dysfunction

Physiological data at different time points are summarized in Table 1. The body weights of *apo E*^−/−^ and *apo E*^−/−^*MMP-12*^−/−^ mice on HFD increased at similar rates throughout the experimental period. When mice were switched to HFD, there was an increase in blood glucose concentration, and total cholesterol, triglyceride, HDL, and LDL levels compared to control (*apo E*^−/−^) mice fed regular chow. Systolic blood pressure values did not differ significantly between *apo E*^−/−^ and *apo E*^−/−^*MMP-12*^−/−^ mice at any time point. *Apo E*^−/−^ mice consuming HFD showed increases in 24-h urinary protein excretion and serum creatinine concentration at 6 and 9 months relative to control mice fed normal chow, whereas these values were reduced at the same time points in mice that also lacked *MMP-12*. But, MMP-12 deletion does not affect the distribution of circulating leukocytes in *apo E*^−/−^ and *apo E*^−/−^ *MMP-12*^−/−^ mice (Supplemental table 1).

### HFD induces morphological changes in the mesangial area of the kidney

**Figure 1:**
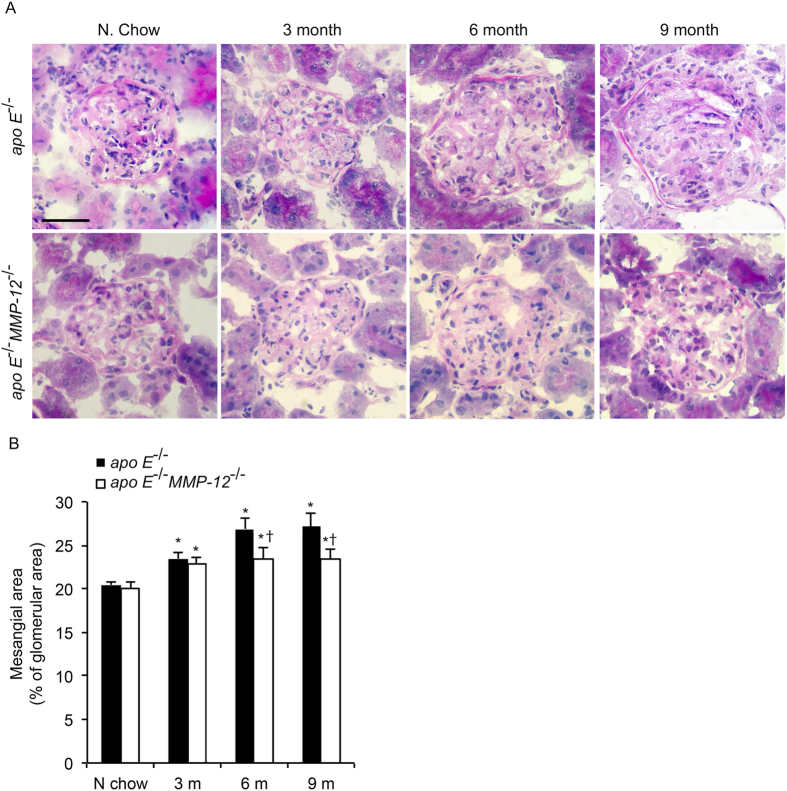
Effects of MMP-12 on glomerular expansion in obese mice. *Apo E*^−/−^ and *apo E*^−/−^*MMP-12*^−/−^ mice and their heterozygote littermates (control) were fed a normal or HFD for 3, 6, and 9 months. (**A**) Representative photomicrographs of pathological glomerular features visualized by PAS staining. Scale bar = 20 μm. (**B**) Quantitative analysis of glomerular surface area expressed as a percentage of glomerular area (n = 10 glomeruli per kidney per animal). Values represent means ± SEM (n = 12 mice per group). *P < 0.05 vs. mice on normal chow; ^†^*P* < 0.05 vs. mice on HFD at the same time point.

The role of MMP-12 in HFD-induced kidney injury was investigated by characterizing morphologic changes in the mesangial area of the kidney by PAS staining. The glomerular matrix in each group was quantified and expressed as a percentage of glomerular area. Mice fed HFD showed a significant increase in glomerular matrix area at 3, 6, and 9 months, an effect that was abrogated with concurrent *MMP-12* deficiency (Fig. 1).

### MMP-12 deletion suppresses HFD-induced profibrotic and proinflammatory gene expression in renal glomeruli

**Figure 2:**
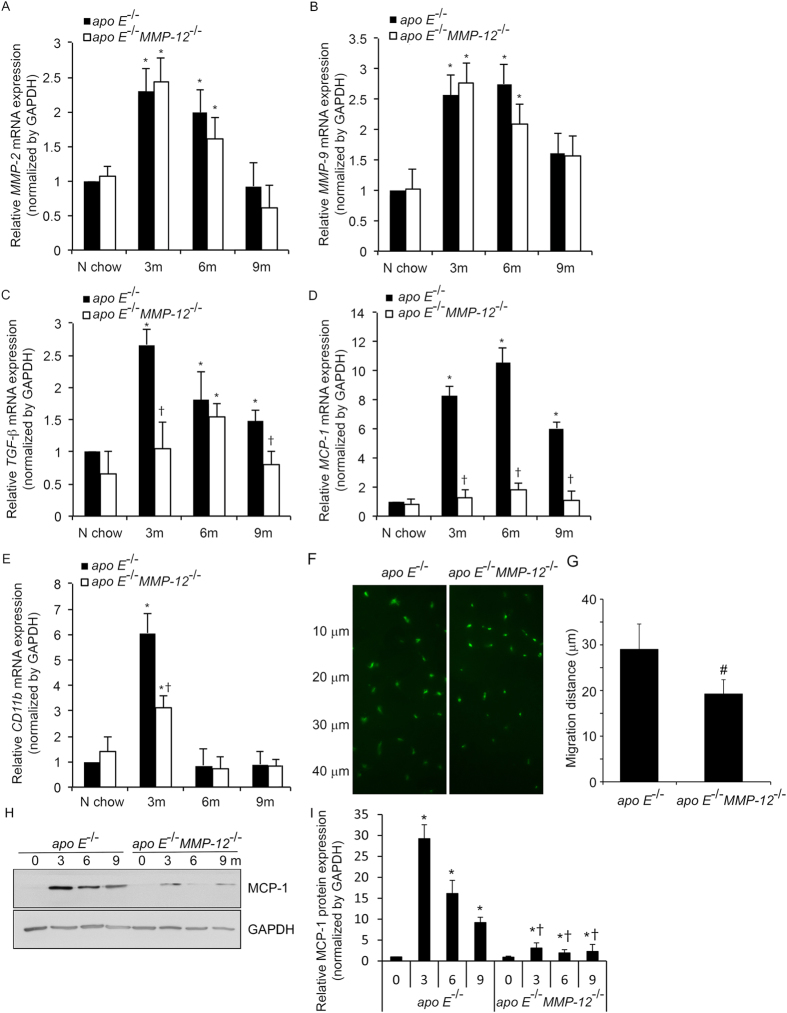
MMP-12 deletion suppresses HFD-induced profibrotic and proinflammatory gene or protein expression in isolated renal glomeruli correlation with macrophage invasion. (**A–E**) The mRNA expression levels of *MMP*-*2* and -*9*, *TGF*-β, *MCP-1*, and *CD11b* were evaluated by qRT-PCR. (**F–G**) Macrophage cells were seeded on the top of collgen IV gel and after 48 hours were fixed and stained by CD68 (green). The images represent vertical position of optical sections captured at 10 μm intervals from top of matrix (0 μm) to 40 μm of invasion depth. (**H,I**) Representative western blot (**H**) and quantification (**I**) of relative MCP-1 protein levels in *apo E*^−/−^ and *apoE*^−/−^*MMP-12*^−/−^ mice fed normal chow or HFD for 3, 6, and 9 months. Values represent means ± SEM (n = 6 per group). *P < 0.05 vs. mice on normal chow; ^†^*P* <0.05 vs. mice on HFD at the same time point; ^#^*P* < 0.05 vs. *apo E*^−/−^ mice.

Pure glomerular RNA was obtained from isolated glomeruli of *apo E*^−/−^ and *apo E*^−/−^*MMP-12*^−/−^ mice and analyzed by qRT-PCR for profibrotic and proinflammatory gene expression using TaqMan probes. Transcript levels of *MMP-2* and *-9* were increased in mice on HFD, but there were no differences between *apo E*^−/−^ and *apo E*^−/−^*MMP-12*^−/−^ mice at any time point (Fig. 2A,B). In contrast, the mRNA level of the profibrotic marker gene *TGF-β*_1_ was increased in glomeruli of HFD *apo E*^*−/−*^ mice at the 3-month time point, whereas *MMP-12* deficiency suppressed this increase at 3 and 9 months (Fig. 2C). HFD is associated with macrophage recruitment in glomeruli^24^; this was evidenced by upregulation of the monocyte/macrophage marker *CD11b* and the chemokine *MCP-1* in the glomeruli of HFD *apo E*^−/−^ mice at 3, 6, and 9 months (Fig. 2D,E). Meanwhile, the concomitant deletion of MMP-12 attenuated the increases in *CD11b* and *MCP-1* expression at these time points. *In vitro* cell invasion assays in 3D collagen IV showed vertical invasion depth of macrophage cells from *apo E*^−/−^*MMP-12*^−/−^ mice less than those from *apo E*^−/−^ mice, which it correlated with MCP-1 protein expression (Fig. 2F–I).

### HFD induces MMP-12 expression in renal glomeruli

**Figure 3:**
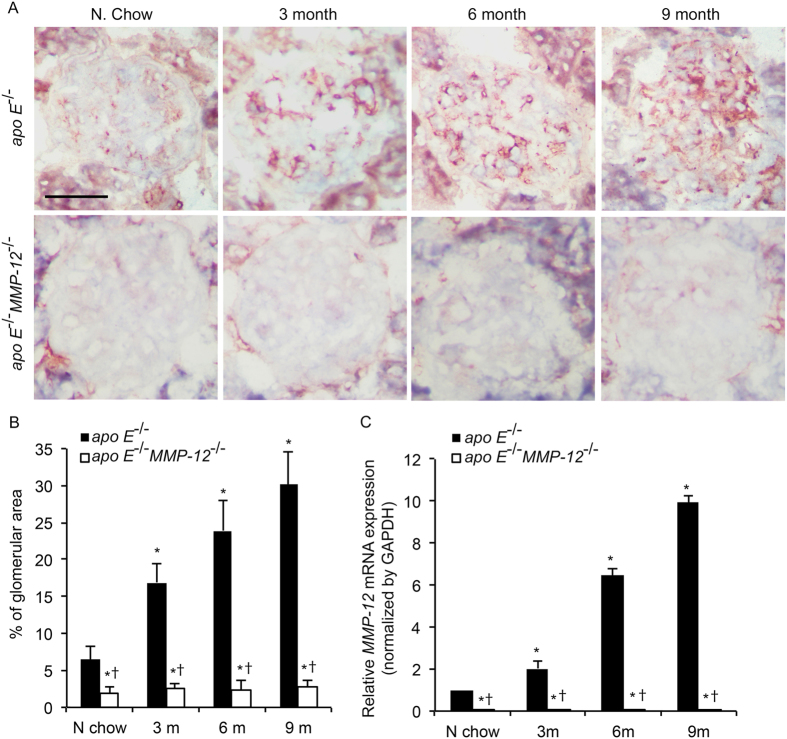
HFD induces MMP-12 expression in renal glomeruli. (**A**) MMP-12 expression in *apo E*^−/−^ and *apo E*^−/−^*MMP-12*^−/−^ mice fed normal chow or HFD for 3, 6, and 9 months was detected by immunohistochemistry. Scale bar = 20 μm. (**B**) Quantitative analysis of MMP-12 expression calculated as a percentage of positive staining within the glomerular area (10 glomeruli per kidney per animal, n = 12 per group). (**C**) HFD-induced *MMP-12* mRNA expression in isolated renal glomeruli, as evaluatedby qRT-PCR. Values represent means ± SEM (n = 6) in each group. *P < 0.05 vs. mice on normal chow; ^†^*P* < 0.05 vs. mice on HFD at the same time point.

Given the broad substrate specificity of MMP-12, we investigated whether MMP-12 modulates glomerular disease progression induced by HFD. Significant MMP-12 immunostaining was detected in the renal glomeruli of HFD *apo E*^−/−^ mice compared to those fed normal chow at 3, 6, and 9 months, whereas *MMP-12* expression was undetectable in glomeruli of *apo E*^−/−^*MMP-12*^−/−^ mice (Fig. 3A,B). To determine whether *MMP-12* is induced as a function of glomerular disease progression, glomerular RNA was analyzed by qRT-PCR. *MMP-12* mRNA level increased with time; expression was absent in *apo E*^−/−^*MMP-12*^−/−^ and only weakly detected in *apo E*^−/−^ mice fed normal chow; however, the expression levels increased markedly 3, 6, and 9 months after *apo E*^−/−^ mice were switched to HFD, suggesting that *MMP-12* expression is induced over the course of glomerular disease progression by consumption of HFD (Fig. 3C).

### MMP-12 deletion attenuates HFD-induced macrophage infiltration in renal glomeruli

**Figure 4:**
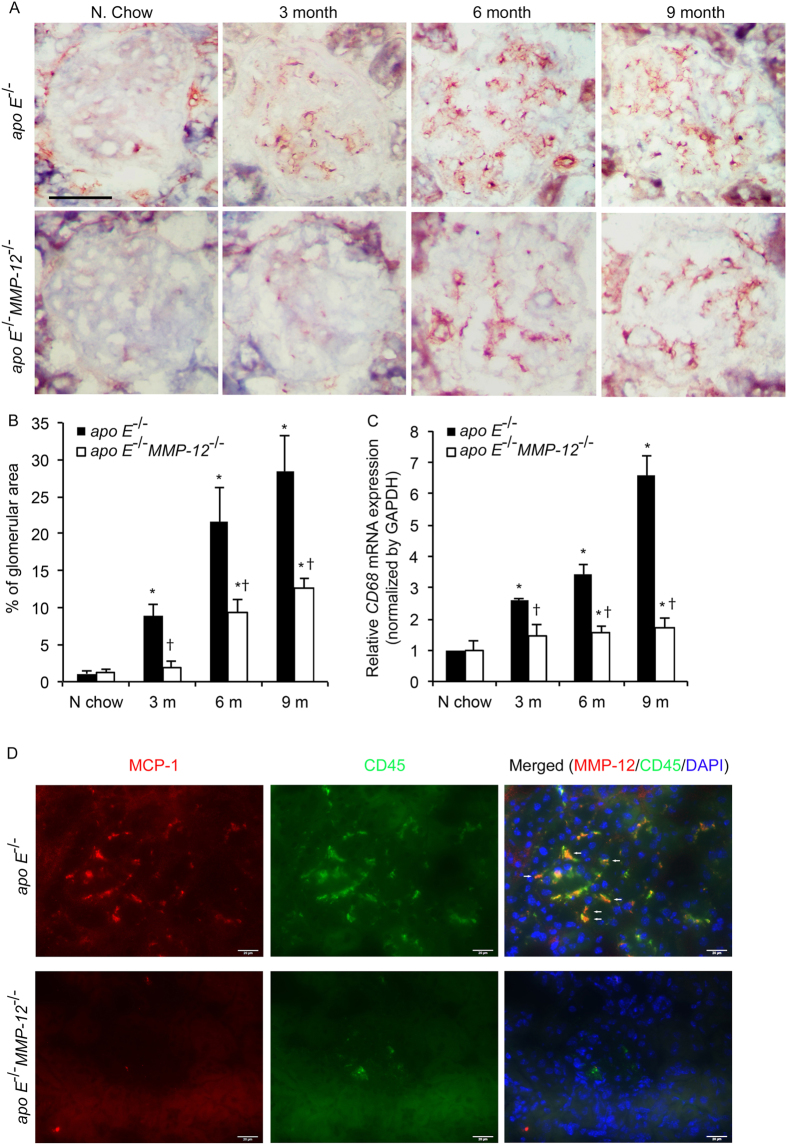
MMP-12 deletion suppresses HFD-induced macrophage infiltration into renal glomeruli. (**A**) Expression of the macrophage marker CD68 in *apo E*^−/−^ and *apo E*^−/−^*MMP-12*^−/−^ mice fed normal chow or HFD for 3, 6, and 9 months. Scale bar = 20 μm. (**B**) Quantitative analysis of glomerular CD68 expression calculated as a percentage of positive staining within the glomerular area (10 glomeruli per kidney per animal, n = 12 per group). (**C**) HFD induced CD68 mRNA expression in isolated renal glomeruli evaluated by qRT-PCR. Values represent means ± SEM (n = 6 in each group). *P < 0.05 vs. mice on normal chow; ^†^*P* < 0.05 vs. mice on HFD at the same time point. (**D**) Co-localization of MCP-1 and CD45 in renal glomeruli. Immunofluorescent staining show MCP-1 (red) and CD45 (green) in tissue sections from in *apo E*^−/−^ and *apo E*^−/−^*MMP-12*^−/−^ mice fed HFD for 3 months. The co-expression of MCP-1 in CD45+ cells was observed in *apo E*^−/−^ mice glomeruli (arrow). Nuclei are shown counterstained with DAPI (blue). Scale bar = 20 μm.

To examine the role of MMP-12 in renal inflammation, macrophage infiltration into glomeruli was investigated. MMP12 deletion decreased the ability of the macrophage infiltration using *in vitro* cell invasion assays (Fig. 2F,G). The expression of the macrophage marker CD68 was upregulated in glomeruli at 3, 6, and 9 months in HFD *apo E*^−/−^ mice relative to those fed normal chow (Fig. 4A,B). This increase in macrophage infiltration induced by HFD was abrogated by *MMP-12* deletion. *CD68* mRNA level increased in glomeruli at all the time points in HFD *apo E*^−/−^ but not *apo E*^−/−^*MMP-12*^−/−^ mice (Fig. 4C). Given that similar trends were observed for *MCP-1* and *CD11b* mRNA levels (Fig. 2D,E) and MCP-1 protein levels (Fig. 2H,I), these results indicate that the loss of MMP-12 function inhibits renal inflammation in glomerular disease progression resulting from HFD. Immunofluorescent staining shows MCP-1 secrete increasing in CD45 (macrophage or leukocytes marker) positive cell in tissue sections from in *apo E*^−/−^ mice fed HFD for 3 months (Fig. 4D).

**Figure 5:**
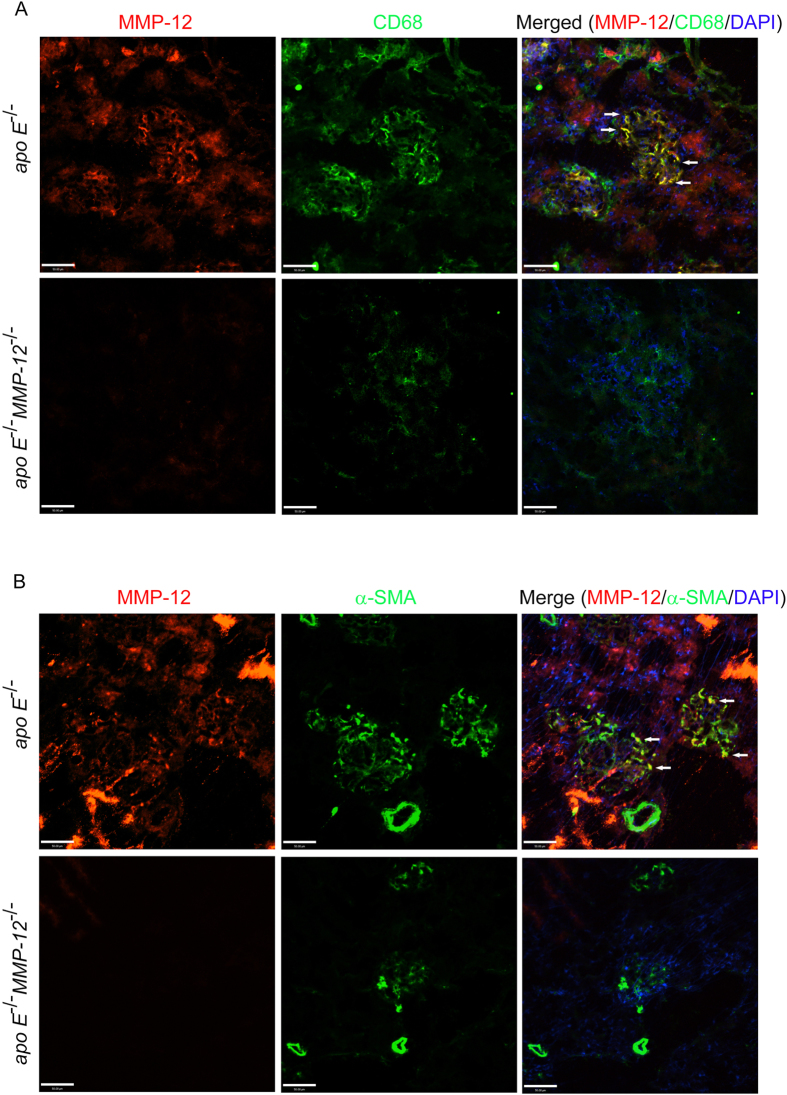
Co-localization of MMP-12 and CD68 or α-SMA in renal glomeruli. (**A**) Confocal images of MMP-12 (red) and CD68 (green) immunoreactivity in tissue sections from *apo E*^−/−^ and *apo E*^−/−^*MMP-12*^−/−^ mice maintained on HFD for 6 months. Most CD68+ infiltrated macrophages coexpressed MMP-12 in *apo E*^−/−^ but not *apo E*^−/−^*MMP-12*^−/−^ mice (arrow). Nuclei were counterstained with DAPI. Scale bar = 50 μm. (**B**) Co-localization of MMP-12 and α-SMA expression in renal glomeruli. Confocal images show MMP-12 (red) and α-SMA (green) immunoreactivity in tissue sections from *apo E*^−/−^ and *apo E*^−/−^*MMP-12*^−/−^ mice fed HFD for 6 months. The co-expression of MMP-12 in α-SMA+ mesangial cells was observed in *apo E*^−/−^ glomeruli (arrow). Nuclei are shown counterstained with DAPI (blue). Scale bar = 50 μm.

To determine whether the MMP-12 detected in glomeruli of mice on HFD is attributable to infiltrated macrophages, tissue sections from HFD *apo E*^−/−^ and *apo E*^−/−^*MMP-12*^−/−^ mice at the 6-month time point were probed for MMP-12 and CD68 expression by immunofluorescence. The percentage of cells expressing both proteins was increased in glomeruli of *apo E*^−/−^ mice on HFD; however, MMP-12 immunostaining was not detected and few macrophages were present in *apo E*^−/−^*MMP-12*^−/−^ glomeruli. Most of the infiltrated (CD68+) macrophages co-expressed MMP-12 in *apo E*^−/−^ samples (Fig. 5A). Glomerular mesangial cells, a type of modified smooth muscle cell, are activated by local injury and synthesize ECM, which affects the progression of renal dysfunction in humans and experimental models of renal diseases^25^,^26^. In *apo E*^−/−^ and *apo E*^−/−^*MMP-12*^−/−^ glomeruli a subset of MMP-12-positive cells expressed smooth muscle α-actin (α-SMA), indicating that they were activated mesangial cells (Fig. 5B).

### MMP-12 deletion suppresses HFD-induced glomerular fibrosis

**Figure 6:**
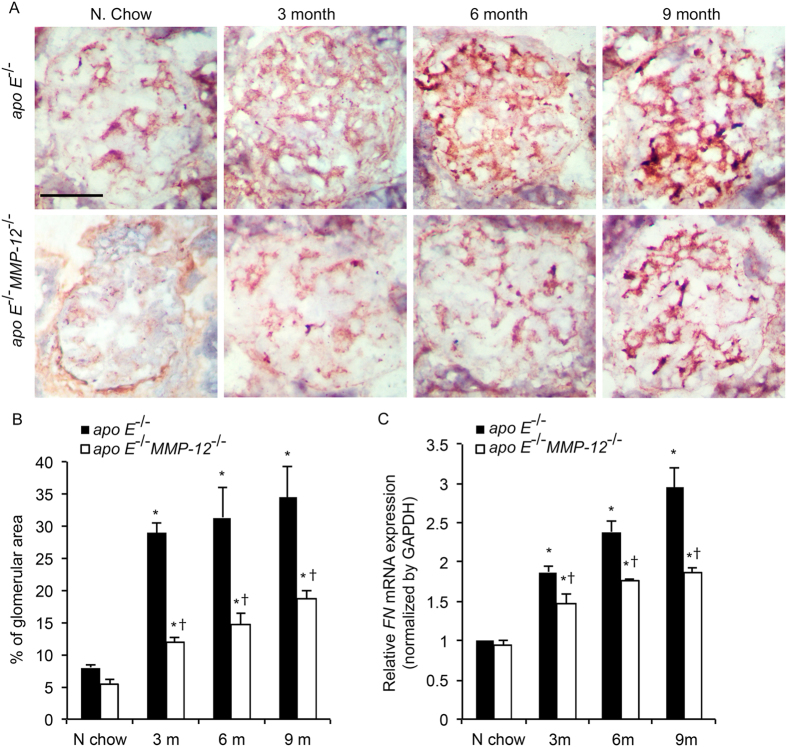
MMP-12 deletion inhibits HFD-induced FN expression in renal glomeruli. (**A**) FN expression in *apo E*^−/−^ and *apo E*^−/−^*MMP-12*^−/−^ mice fed normal chow or HFD for 3, 6, and 9 months was detected by immunohistochemistry. (**B**) Quantitative analysis of FN expression in glomeruli calculated as a percentage of positive staining within the glomerular area (10 glomeruli per kidney per animal, n = 12 per group). (**C**) HFD induced *FN* mRNA expression in isolated renal glomeruli evaluated by qRT-PCR. Values represent means ± SEM (n =  = 6 per group). *P < 0.05 vs. mice on normal chow; ^†^*P* < 0.05 vs. mice on HFD at the same time point.

**Figure 7:**
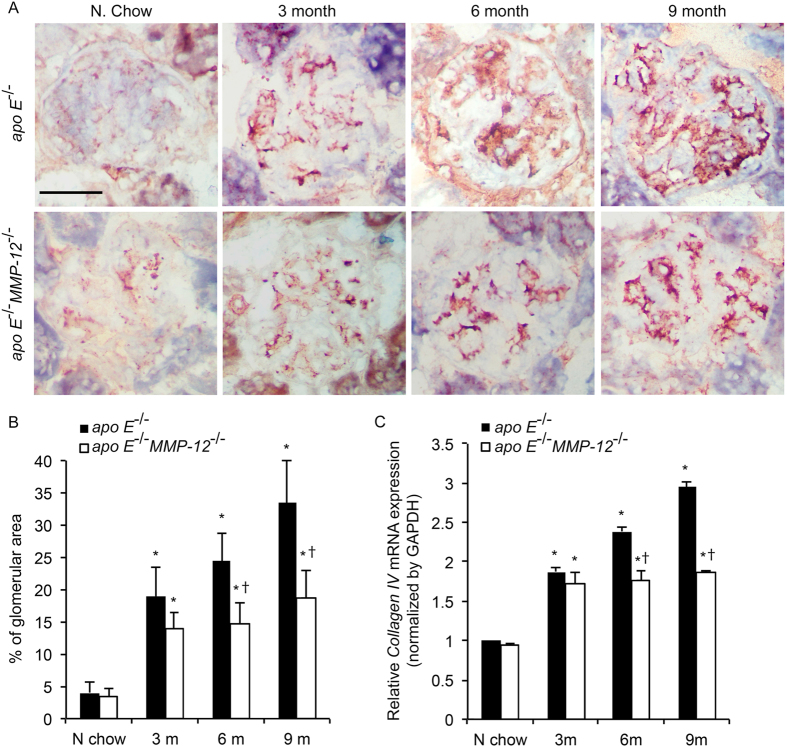
MMP-12 deletion abrogates HFD-induced collagen IV expression in renal glomeruli. (**A**) Collagen IV expression in *apo E*^−/−^ and *apo E*^−/−^*MMP-12*^−/−^ mice fed normal chow or HFD for 3, 6, and 9 months was detected by immunohistochemistry. (**B**) Quantitative analysis of collagen IV expression in glomeruli calculated as a percentage of positive staining within the glomerular area (10 glomeruli per kidney per animal, n = 12 per group). (**C**) HFD-induced *collagen IV* mRNA expression in isolated renal glomeruli, as evaluated by qRT-PCR. Values represent means ± SEM (n = 6 per group). *P < 0.05 vs. mice on normal chow; ^†^*P* < 0.05 vs. mice on HFD at the same time point.

Glomerular matrix accumulation is the hallmark of HFD-induced CKD^3^. To determine whether MMP-12 is involved in this process, the mRNA and protein expression of FN and collagen IV was detected in glomeruli by qRT-PCR and immunohistochemistry, respectively. Consuming HFD induced the expression of both FN and collagen IV at all the time points in *apo E*^−/−^ mice (Figs 6 and 7), whereas the absence of *MMP-12* (in *apo E*^−/−^*MMP-12*^−/−^ mice) was associated with the downregulation of both factors at the mRNA and protein levels.

### MMP-12 deletion suppresses HFD-induced oxidative stress

**Figure 8:**
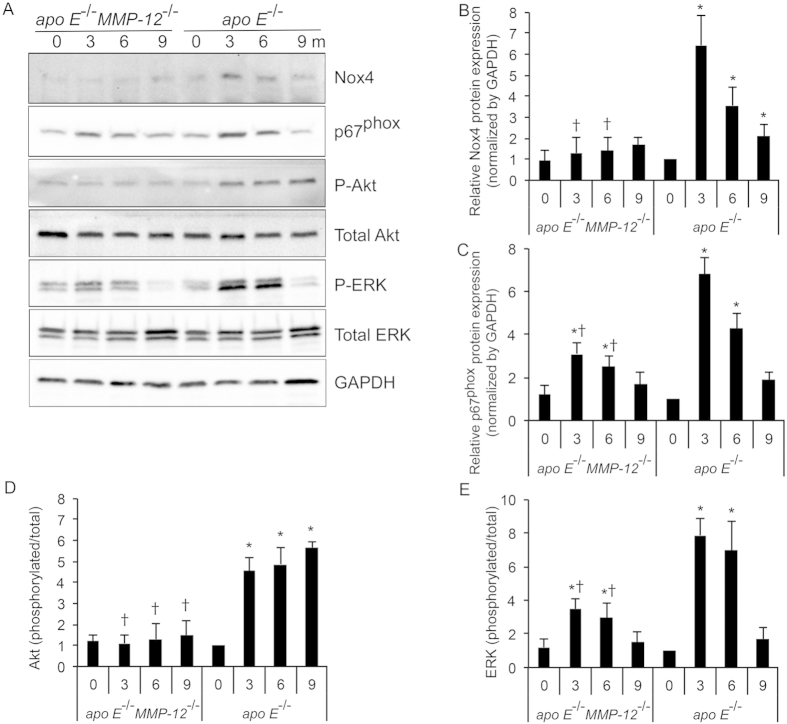
MMP-12 deletion suppresses HFD-induced Nox4 and p67^phox^ and activation of downstream signaling in glomeruli. (**A**) Representative Western blot and quantification (**B–E**) of relative Nox4 and p67^phox^ protein levels, and HFD-induced MAPK p44/42^Thr202/Tyr204^ and Akt^Ser473^ phosphorylation in *apo E*^−/−^ and *apo E*^−/−^*MMP-12*^−/−^ mice fed normal chow or HFD for 3, 6, and 9 months. Values represent means ± SEM (n = 6 per group). *P < 0.05 vs. mice on normal chow; ^†^*P* < 0.05 vs. mice on HFD at the same time point.

Oxidative stress plays a critical role in the pathophysiology of CKD^18^; Nox4 is a major source of ROS in diabetic nephropathy (DN)^27^ and mediates the differentiation of fibroblasts into myofibroblasts, an essential step in renal fibrogenesis^5^,^28^. The subunit p67^*phox*^ is a key activator of Nox2 (gp91^phox^)-expressing NADPH oxidase in phagocytes^29^. To determine the role of MMP-12 in oxidative stress and fibrosis progression in the kidney, Nox4 and p67^phox^ expression in glomeruli was detected by western blotting. HFD caused an increase in Nox4 and p67^phox^ expression in glomeruli of *apo E*^−/−^ but not *apo E*^−/−^*MMP-12*^−/−^ mice at 3, 6, and 9 months, an effect that was abrogated by MMP-12 deletion (Fig. 8).

**Figure 9:**
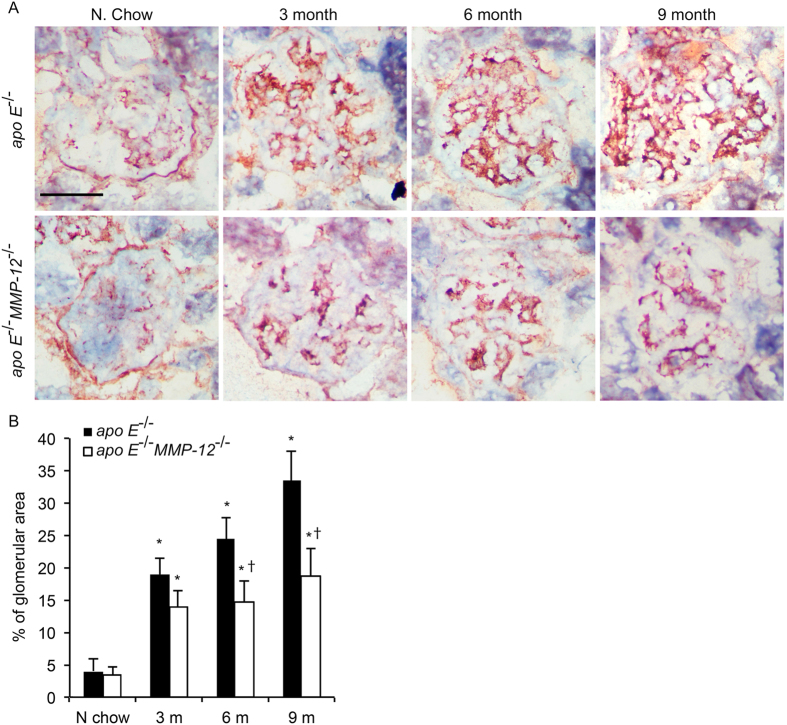
MMP-12 deletion attenuates HFD-induced oxidative damage in renal glomeruli. Oxidative damage in *apo E*^−/−^ and *apo E*^−/−^*MMP-12*^−/−^ mice fed normal chow or HFD for 3, 6, and 9 months was assessed by detecting nitrotyrosine expression by immunohistochemistry. (**B**) Quantitative analysis of glomerular nitrotyrosine level expressed as a percentage of positive staining within the glomerulararea (10 glomeruli per kidney per animal). Values represent means ± SEM (n = 12 mice per group). *P < 0.05 vs. mice on normal chow; ^†^*P* < 0.05 vs. mice on HFD at the same time point.

The serine-threonine kinase Akt/ PKB and the mitogen-activated protein kinase family member-ERK1/2 are activated by phosphorylation, and both play critical roles in cell growth and hypertrophy as well as matrix expansion^3^,^30^. To assess whether these kinases are involved in HFD-induced glomerular disease progression, their phosphorylation status was examined in *apo E*^−/−^ and *apo E*^−/−^*MMP-12*^−/−^ mice. Akt/PKB and ERK1/2 phosphorylation was markedly increased in glomeruli of HFD *apo E*^−/−^ mice at each time point, but this effect was abolished by loss of *MMP-12*. Consistent with these findings, immunostaining for nitrotyrosine, a marker of oxidative damage, was increased in glomeruli of *apo E*^−/−^ mice but reduced in *apo E*^−/−^*MMP-12*^−/−^ mice (Fig. 9). These findings demonstrate that *MMP-12* deletion attenuates Nox4/p67^phox^-associated oxidative stress induced by HFD and the activation of downstream signaling pathways that lead to fibrogenesis in glomeruli.

## Discussion

MMP-12-induced degradation of the basement membrane—which is mainly composed of collagen IV and LN—can undermine the integrity of the renal parenchyma and thereby facilitate inflammatory macrophage infiltration, leading to CKD^22^,^23^. The present study demonstrates that MMP-12 produced by macrophages infiltrating into glomeruli may be a major factor in glomerular fibrogenesis and inflammation resulting from HFD. These effects were attenuated and renal function was preserved in *apo E*^−/−^*MMP-12*^−/−^ relative to *apo E*^−/−^ mice. Characteristic features of HFD-induced obesity in kidney disease include glomerular hypertrophy, glomerular basement membrane thickening, endothelial and podocyte dysfunction, mesangial matrix accumulation, renal inflammation, interstitial fibrosis, and a progressive decrease in kidney function leading to end-stage renal disease^3^,^11^,^31^. The present data showed that glomerular *MCP-1* mRNA expression was increased by nearly 10-fold in *apo E*^−/−^ mice consuming HFD for 6 months as compared to mice fed normal chow, while the increase was attenuated in *apo E*^−/−^ mice that also lacked *MMP-12*. The upregulation of MCP-1 expression could contribute to subsequent macrophage or leukocytes recruitment and the release of proinflammatory (CD11b) and profibrotic factors (TGF-β) in glomeruli^24^,^32^.

MMP-12 expression was significantly increased from 3 to 9 months after initiation of HFD and colocalized with that of CD68 in *apo E*^−/−^ glomeruli, indicating that MMP-12 is mainly produced by macrophages infiltrating into injured tissues that are undergoing remodeling. Although the molecular basis of this process is not fully understood, MCP-1 secreted from mesangial or endothelial cells or infiltrating CD8-positive lymphocytes or macrophages induces glomerular macrophage accumulation, and other members of the MMP family such as MMP-2 and -9 degrade ECM constituents in the kidney and are thus implicated in several renal disease models^9^,^24^,^31^. MMP-12 deletion attenuated HFD-induced *MCP-1* but not *MMP-2* or *-9* expressions, suggesting that MMP-12 is a major but not the sole factor responsible for glomerular injury.

MMP-12 degrades ECM components comprising the glomerular basement membrane^33^; elastin in the basement membrane of Bowman’s capsule is a major MMP-12 substrate^33^,^34^. The induction of MMP-12 expression and consequent accumulation of macrophages in *apo E*^−/−^ relative to *apo E*^−/−^*MMP-12*^−/−^ mice on HFD suggest that MMP-12 secreted by macrophages contributes to the rupture of the basement membrane of glomeruli or Bowman’s capsule, resulting in macrophage infiltration into Bowman’s space. Similar results were observed in a study of antiglomerular basement membrane disease in rats in which administration of an antibody against MMP-12 reduced glomerular macrophage accumulation, preserved glomerular structure, and protected against renal dysfunction^31^.

MMP-12 modulates fibrosis in injured tissue; for instance, MMP-12-null mice do not develop pulmonary fibrosis in the Fas-induced model of acute lung injury^12^, and in corneal wound healing, MMP-12 contributes to the recruitment of inflammatory cells and inhibits the angiogenic response^14^,^35^. In the present study, HFD stimulated macrophage infiltration and increased TGF-β, FN, and collagen IV expression in *apo E*^−/−^ mice. TGF-β is a potent, well-characterized mediator of tissue fibrosis, including renal glomerulosclerosis^36^. TGF-β upregulation has been shown to increase collagen accumulation *in vitro*, which contributes to progressive kidney disease^16^,^37^. As the major component of basement membranes, collagen IV exists in a highly complex superstructure form that not only acts as a scaffold for structural support but also has a signaling function that can potentially contribute to glomerular matrix accumulation and mesangial expansion^38^,^39^. FN is overexpressed in several glomerulopathies, including DN; indeed, FN is also one of the first ECM proteins that are upregulated in the early stages of the disease. FN dimers bind α_5_β_1_ integrins and the ensuing FN-FN interactions form fibrils that assemble into FN matrices, which interact with cells to induce the formation of additional matrices, a potentially important phenomenon in chronic and progressive renal diseases such as DN^9^,^40^,^41^. The stimulatory effect of high glucose on α_5_β_1_ integrin-mediated FN matrix assembly could accelerate the accumulation of FN and induce collagen IV deposition by mesangial cells in DN^4^. Our results show that HFD-induced glomerular fibrosis is associated with CD68-positive macrophage infiltration mediated by various chemokines such as MCP-1 secreted by resident glomerular cells. MMP-12 shows renoprotective effects partly via regulation of macrophage infiltration and accumulation in the cortex, which occurs concurrently with matrix deposition and fibrosis. In the future, more studies will test the effect of macrophage-specific MMP12-deficiency, or the contribution from infiltrating leukocytes that could have been address with different models (cell-specific or bone marrow transplant). Moreover, other cell sources of MMP-12, such as fibrocytes, which might be secondarily induced by macrophages, will be addressed.

Oxidative stress results from an imbalance between the generation and scavenging of ROS such as superoxide anion (O_2_^−^) or hydrogen peroxide (H_2_O_2_). Increased ROS generation production is a hallmark of kidney diseases^19^; however, ROS also act as transcriptional regulators, for instance in the production of profibrotic growth factors such as TGF-β^18^,^19^. The induction of Nox4 expression by TGF-β has been implicated in basal ROS production in the kidney and in pathologic conditions such as DN and CKD. NADPH oxidase isoforms are comprised of different core catalytic subunits that assemble on the membrane upon activation to generate O_2_^−19^. The cytosolic subunit p67^phox^ contains an NADPH-binding site and activates the enzyme by facilitating the transfer of electrons to the flavin center of cytochrome *b*. Absence or dysfunction of p67^phox^ results in impaired phagocyte O_2_^−^ production leading to salt-sensitive hypertension and renal oxidative stress and injury^29^.

In this study, *apo E*^*−/−*^ but not *apo E*^−/−^*MMP-12*^−/−^ mice maintained on HFD exhibited enhanced Nox4 and p67^phox^ expression in glomeruli; the consequent generation of ROS may play a role in renal oxidative stress and kidney injury. *MMP-12* deletion inhibited HFD-induced oxidative stress and downstream phosphorylation of Akt/PKB and ERK1/2 signaling pathways in glomeruli. A possible explanation for this is that TGF-β produced by infiltrating macrophages and upregulated Nox4 expression may induce free radical formation, thereby increasing renal oxidative stress. MMP-12 deletion did not completely block macrophage infiltration, indicating that factors other than MMP-12 such as MMP-2 or -9 contribute to renal oxidative stress and glomerular injury.

The results support our hypothesis that MMP-12 modulates HFD-induced glomerular fibrogenesis and inflammation in a mouse model of obesity. Although MMP12 clearly interacts with other conditions induced by HFD in the model (i.e. higher cholesterol and lipoproteins, which are lower in HFD-fed B6 mice, and concurrent endothelial cell dysfunction or permeability in the glomerulus), but MMP-12 produced by macrophages infiltrating into the glomeruli contributes to the degradation of collagen IV and FN, inducing a renal oxidative stress crescent in Bowman’s space, which is a major factor in the development of renal fibrogenesis and inflammation. Based on these findings, specific regulators of MMP-12 can potentially be used for the treatment of crescentic inflammation and glomerular fibrogenesis in CKD.

## Methods

### Animal model and glomerular isolation

Animal care and treatment procedures were conducted in accordance with the guidelines of the Institutional Animal Care and Use Committee of Hebei Medical University (Shijiazhuang, China), and conformed to the National Institutes of Health Guide for Care and Use of Laboratory Animals. The role of MMP-2 in HFD-induced glomerular fibrogenesis and inflammation was examined in mice lacking *apo E* alone or in conjunction with *MMP-12* deficiency. Single mutants of each gene in a C57BL/6J background were purchased from Jackson Laboratory (Bar Harbor, ME, USA); *apo E*^−/−^*MMP-12*^−/−^ mice were generated by breeding male and female double heterozygotes (*apo E*^+/−^*MMP-12*^+/−^). The *apo E*^−/−^ littermates served as controls. Ninety six male mice were housed in a temperature- and humidity-controlled room on a 12:12 h light/dark cycle and had free access to standard chow that was replaced after 6 weeks by HFD consisting of 1.25% cholesterol and 15.8% fat (TD 90221; Harlan Teklad, Madison, WI, USA) for 3, 6, and 9 months (n = 12 per group). Animal care and treatment followed the guidelines of the Institutional Animal Care and Use Committee of Yale University. At each time point, blood pressure was measured using the Non-Invasive blood pressure system (Kent Scientific Corp, Torrington, CT, USA) prior to anaesthetization with ketamine (60 mg/kg) and xylazine (15 mg/kg) by intramuscular injection, and blood samples were collected from the inferior vena cava to measure blood glucose, total cholesterol, triglyceride, and low- and high-density lipoprotein (LDL and HDL, respectively) levels. The right kidney of each mouse was dissected, rinsed with cold saline, and placed in Tissue-Tek O.C.T. compound (Sakura Finetek USA Inc., Torrance, CA, USA), then snap-frozen in liquid nitrogen and stored at −80 °C until the time of analysis. The renal cortex of the left kidney from each mouse was cut into small pieces, and glomeruli were isolated using the mechanical graded sieving technique. The purity of the final glomerulus suspension was determined by phase contrast microscopy^25^. On average, tubular contamination was <5%. The suspension was used for protein and RNA isolation.

### Histological analysis and immunohistochemistry

Frozen kidney sections (5-μm thick) that were collected on slides were fixed and processed by hematoxylin and eosin and periodic acid schiff (PAS) staining (Sigma-Aldrich, St. Louis, MO, USA). The mesangial area was analyzed to determine the percentage of glomerular area (10 glomeruli per kidney per animal) using Image J software (National Institutes of Health, Bethesda, MD, USA). For immunohistochemistry, sections were fixed in pre-cooled acetone (−20 °C) for 5 min; after three washes in phosphate-buffered saline and a 10-min treatment with 3% H_2_O_2_, sections were serum-blocked and incubated with antibodies against MMP-12, CD68 (both from Abcam, Cambridge, MA, USA), FN, collagen IV (Santa Cruz Biotechnology Inc., Santa Cruz, CA, USA), or nitrotyrosine (EMD Millipore, Billerica, MA, USA), followed by incubation with biotinylated anti-rabbit, anti-rat, or anti-mouse secondary antibody (Vector Laboratories, Burlingame, CA, USA), a streptavidin-horseradish peroxidase conjugate, and finally diaminobenzidine substrate for visualization (Vector Laboratories). For glomerular assessment, the percentage of mesangial area stained as glomeruli was quantitated using 10 glomeruli per kidney per animal, using Image J software.

### Immunocytochemistry

Immunocytochemistry was performed according to a previously described method^25^,^42^. Briefly, frozen sections were fixed with pre-cooled acetone (−20 °C) for 5 min and incubated with antibodies against MMP-12, CD68, α-SMA (Sigma-Aldrich), MCP-1(Santa Cruz Biotechnology), or CD45 (Abcam), followed by Cy3-conjugated anti-rabbit IgG for anti-MMP-12 or anti-MCP-1, Alexa Fluor 488-conjugated anti-rat IgG for anti-CD68, or Alexa Fluor 488-conjugated anti-mouse IgG for anti-α-SMA or anti-CD45 (all from Life Technologies, Grand Island, NY, USA). Sections were mounted with ProLong Gold Anti-fade reagent with DAPI (Life Technologies) and images were acquired with an LSM 510 Meta laser scanning confocal microscope using the 20× objective (Zeiss, Jena, Germany).

### Macrophages isolation and *In vitro* cell invasion assays in 3D collagen IV

Macrophages from *apo E*^−/−^ or *apo E*^−/−^*MMP-12*^−/−^ mice were harvested by peritoneal lavage 5 days after intraperitoneal injection of 4% thioglycolate. Cells were collected by centrifugation, and red blood cells were removed by hypotonic lysis^43^. 2 × 10^5^ cells (>95% macrophages) were added on top of a collagen IV gel in a multiwell plate, and after 48 hours the distance of invasion was measured as the average invasion depth of the cells in the selected field. For each experiment, invasion was analyzed in 3 wells and in 6 fields of vertical position view per individual well^44^.

### Leukocyte Isolations

Whole blood (heparin as anticoagulant, 10 U/mL) were taken from mouse heart, and then leukocytes were isolated using Ficoll-Paque Plus (GE Healthcare). The distributions of circulating leukocytes were counted by automatic hematology analyzer (Beckman Coulter).

### RNA isolation and quantitative real-time PCR

Total RNA was isolated from glomeruli with TRIzol reagent (Invitrogen, Carlsbad, CA, USA). Reverse transcription was carried out using the QuantiTect Reverse Transcription Kit (Qiagen, Valencia, CA, USA). Three independent glomerular preparations were analyzed in triplicate by real-time PCR on an ABI Prism 7700 sequence detection system (Applied Biosystems, Foster City, CA, USA). The following probes were designed using the TaqMan gene assay kit (Applied Biosystems) according to the manufacturer’s instructions: Mm00439498_m1 (MMP-2), Mm00442991_m1 (MMP-9), Mm00500554_m1 (MMP-12), Mm01178820_m1 (TGF-β_1_), Mm03047340_m1 (CD68), Mm00434455_m1 (CD11b), and Mm00441242_m1 (MCP-1). Fold changes in expression were calculated by standardizing RNA level to that of *glyceraldehyde-3-phosphate dehydrogenase* (*GAPDH*) in the same sample.

### Western blotting

Protein extracts of isolated glomeruli from each group (n = 6) were lysed in ice-cold RIPA buffer composed of 150 mM NaCl, 50 mM Tris-HCl (pH 8.0), 1.0% NP-40, 0.5% sodium deoxycholate, and 0.1% SDS supplemented with complete proteinase inhibitor (Roche Applied Sciences, Indianapolis, IN, USA) and phosphatase inhibitor cocktails (Sigma-Aldrich, St. Louis, MO). The homogenates were centrifuged at 13,000 × *g* for 15 min at 4 °C, and protein content of the supernatants was determined using Protein Assay Reagent (Bio-Rad Laboratories, Hercules, CA, USA). Equal amounts of protein were resolved by SDS polyacrylamide gel electrophoresis and transferred electrophoretically to an Immun-Blot PVDF membrane (BioRad Laboratories, Hercules, CA). The membranes were probed with antibodies against Nox4 (Santa Cruz Biotechnology Inc.), p67^phox^, phospho-MAPK p44/42^Thr202/Tyr204^, total MAPK p44/42, phospho-Akt^Ser473^, and total Akt (all from Cell Signaling Technology, Danvers, MA, USA), followed by horseradish peroxidase-conjugated anti-rabbit or anti-mouse secondary antibodies (Cell Signaling Technology, Danvers, MA). Protein bands were visualized using a chemiluminescence detection system (PerkinElmer Life Sciences, Boston, MA, USA). GAPDH was used as a loading control. For antibodies against phosphorylated proteins, membranes were stripped using Restore western blot stripping buffer (Thermo Fisher Scientific, Rockford, IL, USA), and probed with antibodies against the total protein. Films were scanned and quantitated using a ChemiDoc MP Imager system (Bio-Rad Laboratories).

### Study approval

All experimental procedures were conducted in conformity with Ethical Committee and Human Investigational Committee of Hebei Medical University (Shijizhuang, China). All animals’ studies were performed under protocols approved by Hebei Medical University Institutional Guidelines for the Care and Use of Laboratory Animals, and conformed to the National Institutes of Health Guide for Care and Use of Laboratory Animals.

### Statistical analysis

Values are expressed as means ± SEM. Differences between groups were assessed with a two-tailed ratio *t*-test (for paired non-parametric values) or two-way ANOVA with a Bonferroni post hoc test (for > two groups). *P* < 0.05 was considered statistically significant.

## Additional Information

**How to cite this article**: Niu, H. *et al.* Matrix metalloproteinase 12 modulates high-fat-diet induced glomerular fibrogenesis and inflammation in a mouse model of obesity. *Sci. Rep.* **6**, 20171; doi: 10.1038/srep20171 (2016).

## Supplementary Material

Supplementary Information

## Figures and Tables

**Table 1:** Biochemical characteristics of *apo E* ^−/−^ and *apo E* ^−/−^ *MMP12* ^−/−^ mice at different time on HFD.

	*apo E*^−/−^				*apo E*^−/−^*MMP-12*^−/−^			
	N. Chow	3m	6m	9m	N. Chow	3m	6m	9m
Body weight (g)	14.4 ± 0.8	24.7 ± 1.6*	42.6 ± 4.3*	68.6 ± 5.5*	13.8 ± 0.7	25.8 ± 2.1*	39.6 ± 4.8*	62.4 ± 5.2*
Blood pressure (mmHg)	125 ± 6	130 ± 9	131 ± 11	129 ± 12	121 ± 7	128 ± 11	133 ± 9	130 ± 9
24-h protein (mg)	6.6 ± 1.1	8.3 ± 0.9	14.7 ± 3.1*	27.4 ± 2.9*	6.3 ± 0.8	7.5 ± 1.2	8.4 ± 2.3#	11.3 ± 3.7*#
Serum creatinine (mol/L)	8.5 ± 1.7	10.5 ± 2.3	23.7 ± 3.6*	44.2±4.8*	8.3 ± 2.2	9.7 ± 0.9	15.6 ± 3.7*#	22.8 ± 3.6*#
Blood glucose (mg/dL)	105.4 ± 8.4	134.4 ± 10.3*	164.2 ± 12.8*	177.3 ± 21.6*	117.4 ± 12.1	138.2 ± 13.4*	154.7 ± 13.4*	165.3 ± 16.4*
Total cholesterol (mmol/L)	11.4 ± 1.1	15.6 ± 0.9	22.8 ± 2.7*	25.4 ± 3.1*	12.1 ± 0.9	14.5 ± 1.7	20.7 ± 2.1*	23.7 ± 2.6*
Triglycerides (mmol/L)	2.1 ± 0.3	2.4 ± 0.2	3.5 ± 0.4*	3.8 ± 0.3*	1.8 ± 0.3	2.2 ± 0.3	3.2 ± 0.2*	3.6 ± 0.2*
HDL (mmol/L)	2.2 ± 0.2	3.1 ± 0.3*	3.8 ± 0.2*	3.7 ± 0.4*	2.4 ± 0.3	2.8 ± 0.3	3.7 ± 0.4*	3.9 ± 0.3*
LDL (mmol/L)	6.4 ± 0.4	8.3 ± 0.5	18.8 ± 1.9*	21.6 ± 3.1*	7.1 ± 0.4	8.1 ± 0.7	17.4 ± 2.2*	18.4 ± 2.7*

Values represent means ± SEM (n = 12 per group). Statistical analyses were performed by two way ANOVA with Bonferroni post hoc test, *P < 0.05 vs mice on normal chow; ^#^*P* < 0.05 vs mice on HFD at the same time point.
