# Supplementary material for: Matrix metalloproteinase 12 modulates high-fat-diet induced glomerular fibrogenesis and inflammation in a mouse model of obesity
Source: Sci Rep. 2016 Jan 29;6:20171. doi: 10.1038/srep20171 (PMC4731752; doi:10.1038/srep20171)
Supplement: Supplementary Information [file srep20171-s1.pdf]

Supplementary information

Matrix metalloproteinase 12 modulates high-fat-diet induced glomerular fibrogenesis and inflammation in a mouse model of obesity

Honglin Niu,[*†] Ying Li,[*†] Haibin Li,[‡] Yanqing Chi,[*†] Minghui Zhuang[§], Tao Zhang,[*†] Maodong Liu,[*†] Lei Nie[‖¶#]

*Department of Nephrology, Third Hospital of Hebei Medical University, Shijiazhuang, 050051, China
[†]Key Laboratory of Kidney Diseases of Hebei Province, Shijiazhuang, 050071, China
[‡]Department of Cardiology, Third Hospital of Hebei Medical University, Shijiazhuang, 050051, China
[§]Department of Nephrology, First Central Hospital of Baoding, Baoding, 071000, China
[‖]Key Laboratory of Medical Biotechnology of Hebei Province and Key Laboratory of Neural and Vascular Biology of Ministry of Education, Hebei Medical University, Shijiazhuang, 050017, China
[¶]Department of Biochemistry and Molecular Biology, College of Basic Medicine, Hebei Medical University, Shijiazhuang, 050017, China
[#]Corresponding Author

Correspondence to:

Lei Nie, Ph.D

Key Laboratory of Medical Biotechnology of Hebei Province
Key Laboratory of Neural and Vascular Biology of Ministry of Education
Department of Biochemistry and Molecular Biology
College of Basic Medicine, Hebei Medical University
361 Zhongshan East Road, Shijiazhuang, 050017, China

Tel: 86-311-86265639

Email: nieleinl@yahoo.com

Supplemental Table 1. Distribution of circulating leukocytes in *apo E* $^{-/-}$ and *apo E* $^{-/-}$*MMP-12* $^{-/-}$ mice

	CD4	CD8	B cells	~~Macrophages~~	NK cells	Neutrophils
apo E$^{-/-}$	11.6±1.9	10.8±2.8	55.4±8.5	1.4±0.4	1.8±0.5	10.7±4.1
apo E$^{-/-}$ *MMP-12*$^{-/-}$	10.6±1.8	11.2±3.5	51.5±9.2	1.1±0.3	1.5±0.6	9.9±3.6
	P=NS	*P*=NS	*P*=NS	*P*=NS	*P*=NS	*P*=NS

All data are % means±SEM of CD45$^{(+)}$ cells. N=6 in each group. *P*-values were calculated by ANOVA. NK cells, natural killer cells; NS, not significant.
